# Genomic Characterisation of Antimicrobial Resistance and Virulence of Animal-Derived *Klebsiella pneumoniae* Isolates from Germany, and Description of a Hypervirulent Strain

**DOI:** 10.3390/antibiotics15060556

**Published:** 2026-05-30

**Authors:** Marwa Bassiouny, Hanka Brangsch, Ivonne Stamm, Peter A. Kopp, Heinrich Neubauer, Lisa D. Sprague

**Affiliations:** 1 Institute of Bacterial Infections and Zoonoses, Friedrich-Loeffler-Institut (FLI), 07743 Jena, Germany; hanka.brangsch@fli.de (H.B.); heinrich.neubauer@fli.de (H.N.); lisa.sprague@fli.de (L.D.S.); 2 Institute of Pharmacy, Friedrich Schiller University Jena, 07745 Jena, Germany; 3 Vet Med Labor GmbH, IDEXX Laboratories, 70806 Kornwestheim, Germany; ivonne-stamm@idexx.com (I.S.); peter-kopp@idexx.com (P.A.K.)

**Keywords:** *Klebsiella pneumoniae*, WGS, antimicrobial resistance, virulence factors, companion animals, Germany

## Abstract

**Background/Objectives**: *Klebsiella* (*K*.) *pneumoniae* is a significant pathogen in both humans and animals. However, data on its occurrence in animals in Germany remain limited. This study aimed to investigate the antimicrobial resistance (AMR) phenotypes, AMR genes, and virulence traits of animal-derived *K. pneumoniae* isolates from Germany. **Methods**: A total of 59 *K. pneumoniae* isolates obtained in 2023 from dogs, cats, horses, cattle, and chickens across 11 German federal states were analysed. Phenotypic antimicrobial susceptibility testing (AST) was performed, and whole-genome sequencing (WGS) was used for genomic characterisation, including detection of AMR genes, virulence-associated genes, sequence types (STs), and plasmid replicons. **Results**: Most isolates (78%) were susceptible to all tested antibiotics, while three isolates were classified as multidrug-resistant (MDR). Resistance was most frequently observed for piperacillin (n = 8) and trimethoprim/sulfamethoxazole (n = 4). Carbapenem resistance was detected in two isolates (one from a dog and one from a cat), and phenotypic colistin resistance in one dog isolate. WGS identified 96 AMR genes across isolates, with 20–42 AMR determinants per isolate, conferring resistance to β-lactams, aminoglycosides, fluoroquinolones, sulfonamides, tetracyclines, trimethoprim, and fosfomycin. Ten extended-spectrum β-lactamase (ESBL)-producing isolates carried genes including *bla*_CTX-M-15_, *bla*_SHV-2_, *bla*_SHV-27_, *bla*_SHV-42_, *bla*_SHV-106_, and *bla*_TEM-158_. Although *fos*A was detected in all isolates, only three exhibited phenotypic resistance to fosfomycin. A total of 52 STs were identified, including high-risk clones. One hypervirulent isolate (ST60) carrying hypervirulence-associated genes *rmp*A and *iro*B was detected. Plasmid replicons were present in 70% of isolates, while plasmid-associated AMR genes were identified in nine isolates. **Conclusions**: This study demonstrates the genomic diversity of *K. pneumoniae* identified in companion animals and highlights the presence of AMR and virulence determinants relevant to a One Health context.

## 1. Introduction

*Klebsiella* (*K*.) *pneumoniae* is a Gram-negative, encapsulated, non-motile, and rod-shaped bacterium belonging to the family *Enterobacteriaceae* [1]. It is an opportunistic pathogen found in the environment, including soil, surface water, and plants, and is also a commensal on the mucosal surfaces of humans and animals [2]. As a member of the ESKAPE pathogens, *K. pneumoniae* is recognised as a major contributor to nosocomial infections [3]. *K. pneumoniae* is classified into two major pathotypes: classical (cKp) and hypervirulent (hvKp). While cKp primarily causes hospital-acquired infections, hvKp can cause community-acquired infections, even in healthy individuals [4]. The pathogenicity of *K. pneumoniae* is derived from multiple virulence determinants, including its polysaccharide capsule, fimbrial adhesins, iron-acquisition systems (siderophores), and the ability to metabolise allantoin [5]. These factors facilitate immune evasion, adhesion, invasion of host tissues, biofilm formation, and persistence across various hosts and environments [6]. Moreover, *K. pneumoniae* can develop resistance to nearly all antibiotic classes through both intrinsic mechanisms, such as chromosomal β-lactamase production, porin alterations that reduce membrane permeability, and efflux pumps, as well as acquired mechanisms mediated by horizontal gene transfer [7,8].

Clinically, *K. pneumoniae* is associated with a wide range of human infections, including pneumonia, septicemia, urinary tract infections, and liver abscesses, with particularly high mortality among immunocompromised individuals [9]. In animals, *K. pneumoniae* has been isolated from various animal species [10]. It is mainly associated with mastitis in cattle [11] and goats [12], bacteremia in calves [13], and endometritis and metritis in mares [14], as well as urinary tract infections and pneumonia in companion animals such as dogs and cats [15].

In Germany, several nationwide outbreaks of *K. pneumoniae* in humans have been reported [16,17,18]. In animals, *K. pneumoniae* has been identified in companion animals, including rabbits and horses, as well as in pigs, cattle, and broilers [7,19,20]. Despite ongoing research, our understanding of *K. pneumoniae* in animals remains limited, and only a few studies have employed whole-genome sequencing (WGS) in Germany [10,21]. Therefore, this study aimed to address this gap by using next-generation sequencing (NGS) technology to analyse 59 *K. pneumoniae* isolates from companion and farm animals in Germany, focusing on antimicrobial resistance (AMR) profiles, resistomes, and virulomes.

## 2. Results

### 2.1. Bacterial Identification and Whole-Genome Sequencing (WGS)

**Figure 1:**
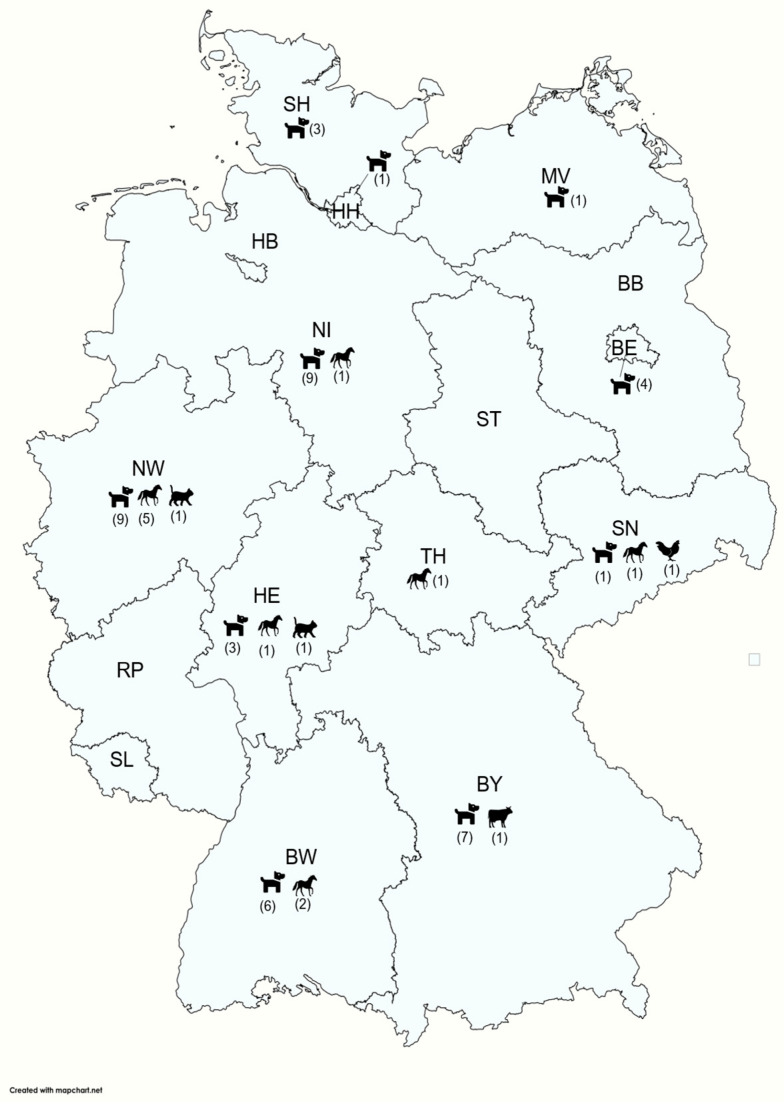
Spatial distribution of 59 *K. pneumoniae* isolates in Germany across animal hosts. The map was generated using MapChart (https://www.mapchart.net/) accessed on 5 December 2025. BW: Baden-Wuerttemberg, BY: Bavaria, BE: Berlin, BB: Brandenburg, HB: Bremen, HH: Hamburg, HE: Hesse, NI: Lower Saxony, MV: Mecklenburg-Western Pomerania, NW: North Rhine-Westphalia, RP: Rhineland-Palatinate, SL: Saarland, SN: Saxony, ST: Saxony-Anhalt, SH: Schleswig-Holstein, TH: Thuringia.

In 2023, a total of 64 bacterial isolates from various companion and farm animals across Germany were initially identified as *K. pneumoniae* by MALDI-TOF MS. WGS subsequently confirmed that 59 of these isolates (92.2%) were *K. pneumoniae*. The remaining five isolates, all originating from dogs, were identified as *K. quasipneumoniae* and were excluded from further analyses in the current study. The confirmed *K. pneumoniae* isolates were obtained from 11 out of the 16 German federal states (Figure 1).

WGS generated an average of 1,214,956 reads per isolate, ranging from 692,582 to 2,713,072. Sequencing coverage averaged 51-fold, with a range of 34-fold to 134-fold. Genome assembly yielded genome sizes between 5.09 and 5.64 Mbp, with an average GC content of 57.35%. The mean N50 value across the 59 assembled genomes was 296,473 bp, ranging from 109,398 bp to 736,814 bp, and the assemblies comprised 24 to 169 contigs (Supplementary Table S1). Additional assembly quality metrics are presented in Supplementary Table S2.

### 2.2. Phenotypic Antibiotic Susceptibility Testing (AST)

AST revealed that 78% of isolates (n = 46) were susceptible to all tested antibiotics. In contrast, three isolates were phenotypically multidrug-resistant (MDR): two from dogs in Saxony and Lower Saxony, and one from a horse in Baden-Wuerttemberg. The highest resistance rate was observed for piperacillin (15.3%; n = 9), followed by trimethoprim/sulfamethoxazole (6.8%; n = 4). Resistance to fosfomycin was identified in three isolates from a horse, a dog, and a cat originating from Baden-Wuerttemberg, Schleswig-Holstein, and North Rhine-Westphalia, respectively.

Imipenem resistance was detected in two isolates, one from a dog and one from a cat originating from Schleswig-Holstein and North Rhine-Westphalia, respectively. Additionally, one dog isolate originating from North Rhine-Westphalia demonstrated colistin resistance (Table 1).

### 2.3. In Silico Detection of AMR and Virulence-Associated Determinants

Genome analysis identified a total of 96 different AMR-associated genes across 59 *K. pneumoniae* isolates, with each isolate carrying between 20 and 42 AMR determinants (Supplementary Table S3). The isolates harboured AMR genes mediating resistance to multiple antibiotic classes, including β-lactams, aminoglycosides, quinolones, tetracyclines, sulfonamides, trimethoprim, phenicols, macrolides, lincosamides and fosfomycin, as well as genes encoding efflux pumps. Three MDR isolates carried the highest number of AMR genes: an ST7682 isolate from a horse in Baden-Wuerttemberg harboured 42 genes, an ST48 isolate from a dog in Lower Saxony carried 38 genes, and an ST147 isolate from a dog in Saxony also harboured 38 genes. AMR genes conferring resistance to quinolones (*qnr*B1, *qnr*B17, *qnr*S1), tetracyclines (*tet*A, *tet*D), sulfonamides (*sul*1, *sul*2), trimethoprim (*dfr*A12 and *dfr*A14), phenicols (*cat*A2 and *flo*R), and macrolides (*mph*A) were detected. The *fos*A gene, which mediates fosfomycin resistance, was present in all isolates, although only three isolates (from a dog, a cat, and a horse) were phenotypically resistant to fosfomycin. In the same context, several aminoglycoside resistance genes including, *aac*(3)-IId, *aac*(3)-IIe, *aac*(6′)-Ib-cr, *aad*A1, *aad*A2, *ant*(3″)-IIa, *ant*(3″)-Ia, *aph*(3″)-Ib and *aph*(6)-Id, were detected, despite all isolates remaining susceptible to amikacin. One canine isolate was colistin-resistant and carried a *crrB*_N141Y mutation, and thirteen isolates were intermediately resistant to colistin and carried the *pmrB*_R256G mutation associated with colistin resistance. Six extended-spectrum β-lactamases (ESBLs) were detected, including *bla*_SHV-2_ (n = 1), *bla*_SHV-27_ (n = 3), *bla*_TEM-158_ (n = 1), *bla*_SHV-42_ (n = 1), *bla*_SHV-106_ (n = 2), and *bla*_CTX-M-15_ (n = 2). No carbapenemase genes were detected in any of the isolates, despite phenotypic carbapenem resistance observed in two isolates.

**Figure 2:**
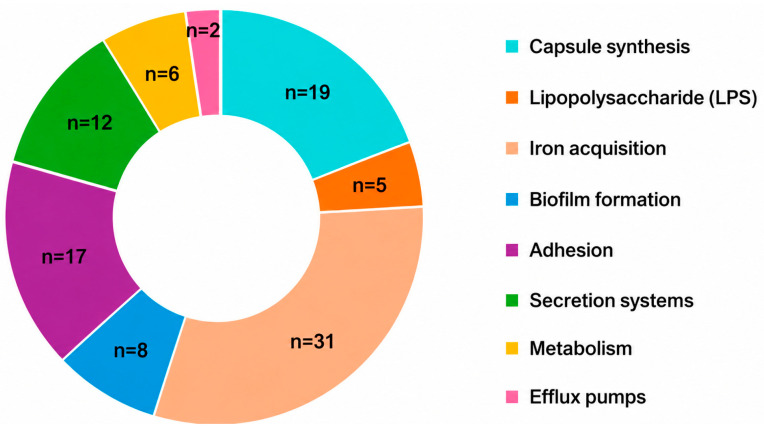
The chart shows the number of virulence-associated genes of different functional categories in *K. pneumoniae* isolates in the present study. n: number of genes.

Genome analysis also revealed a total of 100 virulence-associated genes spanning eight functional categories: capsule synthesis, lipopolysaccharide (LPS), iron acquisition, biofilm formation, adhesion, secretion systems, metabolism, and efflux pumps. Each analysed isolate carried between 48 and 85 virulence-associated genes (Supplementary Table S4). Genes associated with iron acquisition (aerobactin, enterobactin, salmochelin, and yersiniabactin) were the most prevalent (n = 31), followed by capsule biosynthesis regulation (n = 19), and adhesion (n = 17). Efflux-associated genes were the least prevalent (n = 2), as shown in Figure 2.

### 2.4. Identification of Hypervirulent K. pneumoniae

**Figure 3:**
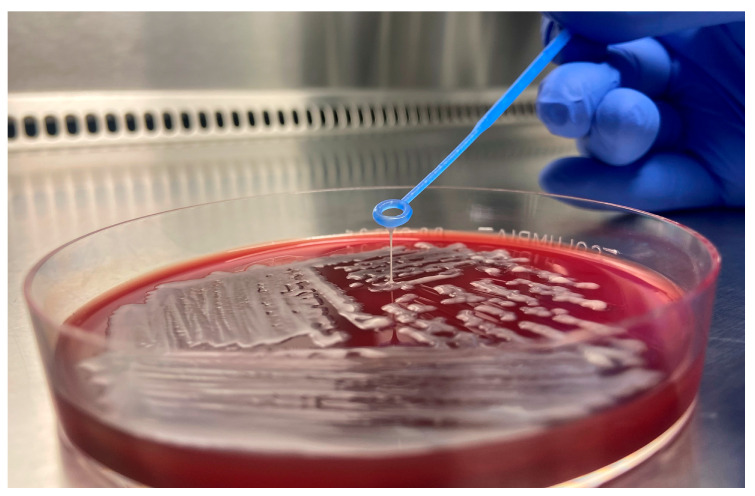
The hypermucoviscous phenotype of *K. pneumoniae* in this study (23Y0130) was demonstrated by a positive string test.

Genome analysis identified two hypervirulence-associated biomarkers, *rmp*A and *iro*B, in a single horse isolate (ST60) from Lower Saxony. The hypervirulent phenotype was confirmed by a positive string test, producing a mucoviscous filament longer than 5 mm (Figure 3). This isolate carried the highest number of virulence-associated genes (n = 85) while harbouring relatively few AMR genes (n = 29). It was susceptible to 15 of the 16 antibiotics tested. Three additional isolates (two from dogs and one from a cat) also yielded a positive string test but did not harbour recognised hypervirulence-associated biomarkers.

### 2.5. Plasmid Content and Plasmid-Associated AMR Genes

**Figure 4:**
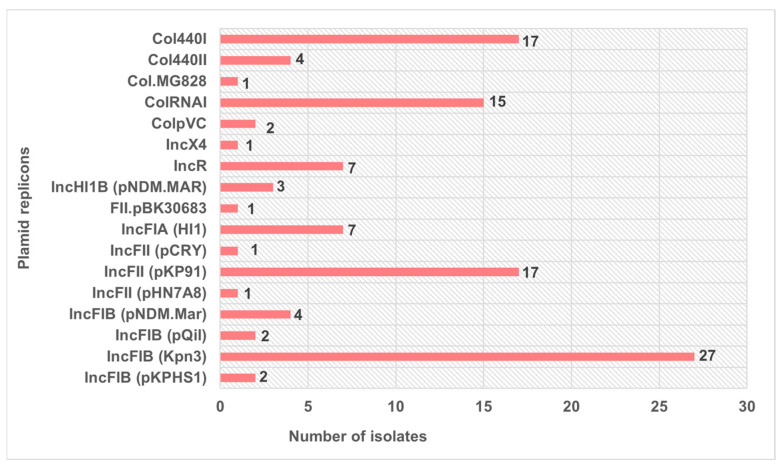
Number of identified plasmid replicons among *K. pneumoniae* isolates of the current study.

Plasmid analysis using PlasmidFinder revealed that approximately 71.2% (n = 42) of the *K. pneumoniae* isolates harboured between one and five plasmid replicons (Supplementary Table S5). A total of 17 plasmid replicons were identified, representing distinct groups, including IncF, IncHI, IncR, IncX4, and Col-type plasmids. The most prevalent replicons were IncFIB (Kpn3), identified in 27 isolates. The IncX4 and Col.MG828 replicons were detected only in the carbapenem-resistant isolate 23Y0206, whereas IncFII.pHN7A8 was present in one of the MDR isolates, 23Y0015. The overall distribution of plasmid replicons among the isolates is summarised in Figure 4.

Further analysis of the plasmids revealed that multiple plasmid contigs carried various AMR genes conferring resistance to several antibiotic classes, including β-lactams, aminoglycosides, tetracyclines, sulfonamides, trimethoprim, phenicols, lincosamides, and macrolides (Supplementary Table S5). Overall, plasmid-borne AMR genes were detected in nine isolates. The three MDR isolates, 23Y0015, 23Y0007, and 23Y0002, harboured the largest number of plasmid-borne AMR genes (14, 10, and 8, respectively). The distribution of plasmid-borne AMR genes identified in the isolates is summarised in Table 2.

### 2.6. Multilocus Sequence Typing (MLST) and CgSNP Analysis

MLST revealed high genetic diversity among the *K. pneumoniae* isolates, identifying 52 distinct STs. The most prevalent ST was ST37, identified in four canine isolates (6.3%) from Lower Saxony (n = 3) and Baden-Wuerttemberg (n = 1), followed by ST200, ST20, ST29, and ST1537, each found in two isolates. The remaining STs were found in one isolate each (Supplementary Table S3). Several internationally recognised high-risk clones were also detected, including ST147, ST101, ST323, and ST45, each represented by a single isolate.

**Figure 5:**
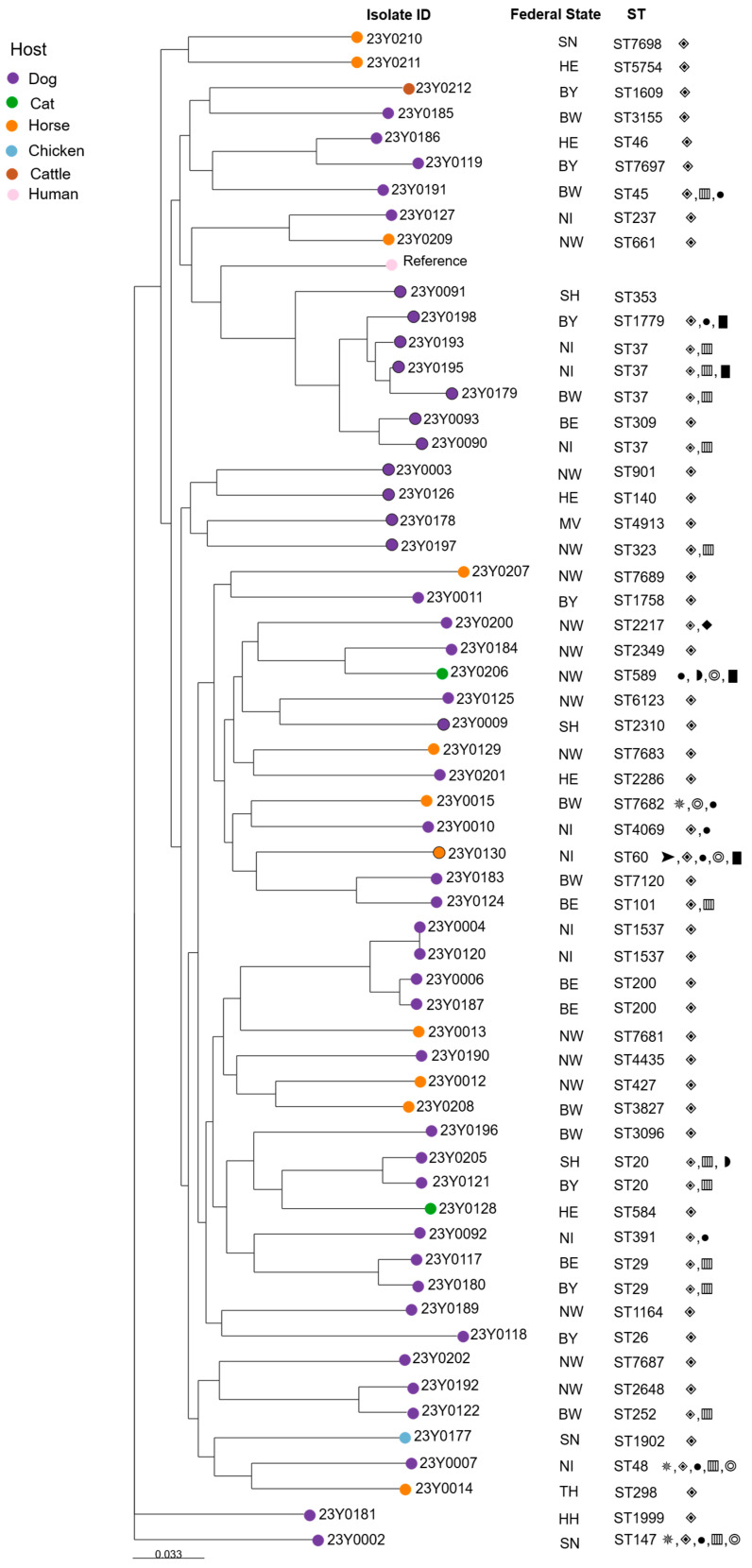
Maximum-likelihood phylogenetic tree of 59 *K*. *pneumoniae* isolates constructed based on core-genome single-nucleotide polymorphisms (cgSNPs). Isolate ID, geographic origin, and sequence type (ST) are indicated for each isolate. The scale bar indicates nucleotide changes per alignment site. Symbols indicate isolate characteristics: ✵ multidrug-resistant isolates; ◆ colistin-resistant isolate; ◗ carbapenem-resistant isolates; ➤ hypervirulent isolate; ▮ isolates with a positive string test; ▥ human-associated STs; ● isolates carrying plasmid-borne antimicrobial resistance genes; ◈ fosfomycin-susceptible isolates despite harbouring the *fos*A gene; ◎ amikacin-susceptible isolates despite harbouring aminoglycoside-resistance genes. German federal state abbreviations: BW: Baden-Wuerttemberg, BY: Bavaria, BE: Berlin, HH: Hamburg, HE: Hesse, NI: Lower Saxony, MV: Mecklenburg-Western Pomerania, NW: North Rhine-Westphalia, SN: Saxony, SH: Schleswig-Holstein, TH: Thuringia.

CgSNP analysis confirmed high genetic diversity among the isolates (Figure 5), except for two isolates from dogs, which were closely related, differing by only eight SNPs (Supplementary Table S6). Both isolates originating from Lower Saxony shared identical antibiotic susceptibility profiles, belonged to ST1537, and carried the same number of virulence genes (n = 64).

## 3. Discussion

*Klebsiella* (*K*.) *pneumoniae* is an important human and animal pathogen, responsible for a wide range of infections and frequently exhibiting resistance to clinically important antimicrobial agents worldwide [22]. It is considered a One-Health pathogen and has been isolated from animals, humans, food, and environmental sources in Germany and worldwide [7,23,24,25]. This study provides a genomic characterisation of 59 *K. pneumoniae* isolates collected from companion and farm animals across 11 German federal states. Following our previously published work [26] focusing on MLST-based ST analysis, including the identification of novel STs, in the current study, we investigated AMR (phenotypic and genotypic), virulence-associated factors, and lineage. The broad geographic distribution observed in the current study highlights the widespread presence of *K. pneumoniae* in animal populations in Germany.

Most isolates originated from North Rhine–Westphalia, and dogs represented the predominant host species in the present study. Hence, no statistical statement could be performed due to missing stratification. Fecal samples constituted the most common source of isolation in the current set of isolates, consistent with previous studies indicating that the gastrointestinal tract serves as a major reservoir for *K. pneumoniae* [27,28]. MLST analysis revealed high genetic diversity, with 52 distinct STs identified [26]; ST37 was the most prevalent, occurring in four isolates. The observed high genetic diversity is likely attributable to the presence of diverse animal hosts, sample types, and geographic distribution. Several human-associated STs were identified, including ST147, ST101, ST37, ST20, ST29, ST252, ST323, ST45, and ST48 [29,30,31,32,33,34,35], indicating genetic overlap between human and animal reservoirs [36]. Several STs, such as ST147, ST20, ST45, and ST323, have previously been reported in animal-derived *K. pneumoniae* isolates in Germany [10], indicating the circulation of clinically relevant lineages across human and animal populations, within a One Health context.

Phenotypic antibiotic susceptibility testing showed high overall susceptibility to tested antibiotics, but three MDR isolates were found. These MDR isolates carried the highest number of AMR genes, consistent with their phenotypic profiles and supporting an association between gene abundance and MDR phenotypes [1]. Carbapenem resistance was detected in two isolates, one from a dog and one from a cat, despite carbapenems not being allowed for use in animals in the European Union [37], suggesting possible transmission from human or environmental sources [38,39]. Notably, no carbapenemase genes were detected among these isolates. In contrast, another study from Germany did not detect carbapenem resistance among animal-derived *K. pneumoniae* isolates [10], indicating that such resistance remains uncommon in animal populations. Similarly, a study conducted in Italy included 3482 genomes from 15 *Klebsiella* species and found no genotypic or phenotypic evidence of non-susceptibility to carbapenems outside the clinical environment [24]. Nevertheless, carbapenemase-producing *K. pneumoniae* has previously been reported in companion animals in Germany [40].

Colistin resistance was detected in a single dog isolate (ST2217). Although the *mcr*-1 gene was absent, the isolate harboured the *crrB*_N141Y mutation, which has previously been associated with colistin resistance [41]. Similar findings of colistin-resistant *K. pneumoniae* isolates from pigs have previously been reported in Germany [10]. Phenotypic fosfomycin resistance was observed in only three isolates, although the presence of the *fos*A gene was detected in all isolates. This genotype and phenotype discrepancy may suggest the presence of silent or non-expressed resistance determinants [42]. Extended-spectrum β-lactamase genes were found in ten isolates, consistent with previous reports from various studies in Germany [20,43], and other European countries [44,45], indicating that these resistance traits are already established and circulating within the companion animal population.

Hypervirulent *K. pneumoniae* (hvKp) has emerged as a distinct pathotype associated with severe community-acquired infections, including liver abscesses, pneumonia, meningitis, and endophthalmitis, even in otherwise healthy individuals, and it is linked to high morbidity and mortality [46]. First described in Taiwan [47], hvKp has since emerged and been disseminated globally with increasing reports worldwide [48].

In Europe, reported hvKp cases have risen in recent years, including detections in both humans and animals, highlighting its growing public health relevance [28,34,49,50,51]. However, its occurrence in animal populations remains underexplored, and the role of animals in hvKp epidemiology is still unclear. In the current study, four isolates exhibited a hypermucoviscous phenotype with a positive string test; however, only a single isolate from a horse (ST60) carried the hypervirulence-associated biomarkers *rmp*A and *iro*B and was therefore described as hvKp. This finding indicates that the string test alone is insufficient for reliable hvKp identification, underscoring the need for combined genomic and phenotypic approaches for accurate identification of hvKp [52,53]. The identified hvKp isolate in this study carried the highest number of virulence-associated genes, while harbouring relatively few AMR genes and remaining susceptible to 15 of 16 tested antibiotics, consistent with classical hvKp profiles [54]. HvKp strains typically have larger, more robust capsules than classical strains. This hypermucoviscous capsule enhances survival by offering extra protection but also acts as a physical barrier in horizontal gene transfer, which could explain the lower frequency of AMR genes in hvKp strains compared to classical strains [46]. The detection of hvKp in companion animals further supports potential circulation at the animal–human interface, although transmission routes and reservoirs remain unclear.

The presence of plasmid replicons carrying AMR genes such as *bla*_CTX-M-15_ and *tet*A demonstrates the crucial role of plasmids in horizontal gene transfer across human, animal, food, and environmental reservoirs [55]. Plasmid analysis revealed that plasmid-borne AMR genes were present in nine isolates, including the MDR strains with the highest plasmid-associated resistance gene burden. Several studies have reported that animal-derived *K. pneumoniae* can carry mobile genetic elements harbouring AMR genes [7,10,56]. Most of the investigated isolates were obtained from dogs, while only a limited number originated from food-producing animals. The narrow host range of the collected isolates represents the main limitation of this study. Therefore, further studies including a larger number of isolates from diverse animal hosts, both companion and food-producing animals across all federal states, as well as a broader application of WGS across diverse sources, are needed to better understand AMR dynamics in *K. pneumoniae*.

## 4. Methods

### 4.1. Bacterial Isolates and Species Identification

A total of 64 putative *K. pneumoniae* isolates were obtained in 2023 from Vet Med Labor GmbH, IDEXX Laboratories (Kornwestheim, Germany), and originated from diagnostic submissions across Germany. The isolates originated from various animal species, including 49 from dogs, 11 from horses, two from cats, one from a chicken, and one from cattle (Table 3). Detailed metadata for each isolate is provided in Supplementary Table S3. Species identity was initially confirmed at the Friedrich-Loeffler-Institut (FLI), Jena, Germany, using matrix-assisted laser desorption/ionization time-of-flight mass spectrometry (MALDI-TOF MS) on an UltrafleXtreme mass spectrometer (Bruker Daltonics, Bremen, Germany), as previously described [57]. Genus and species confirmation was subsequently achieved through WGS data analysis using Kraken2 (v2.0.7_beta) [58].

### 4.2. Antibiotic Susceptibility Testing (AST)

AST was performed for all isolates using the broth microdilution method with the automated MICRONAUT-S (MICRONAUT, MERLIN Diagnostics GmbH, Bornheim-Hersel, Germany), as previously described [59]. MICRONAUT-S MDR MRGN-Screening plates were employed with Mueller-Hinton Broth (Bruker Daltonics GmbH & Co. KG, Bremen, Germany) according to the manufacturer’s instructions. The antibiotic panel included 16 antimicrobial agents representing eight classes: ciprofloxacin, levofloxacin, amikacin, colistin, chloramphenicol, fosfomycin, tigecycline, trimethoprim/sulfamethoxazole, piperacillin, piperacillin/tazobactam, cefotaxime, ceftazidime, ceftazidime/avibactam, ceftolozane/tazobactam, imipenem, and meropenem (Supplementary Table S3). Minimum inhibitory concentrations (MICs) were interpreted according to the recommendations of the Clinical and Laboratory Standards Institute (CLSI) breakpoints for *K. pneumoniae* [60]. MICRONAUT-S software was used for automated interpretation of MIC values according to CLSI breakpoints, following the manufacturer’s instructions. The MICRONAUT-S software automatically classified isolates as susceptible, intermediate, or resistant. Quality control was ensured using standard reference strains: *Escherichia coli* ATCC 25922, *P. aeruginosa* ATCC 27853, and *K. pneumoniae* ATCC BAA-1705.

### 4.3. String Test for Assessment of Hypermucoviscosity

The hypermucoviscous phenotype was determined using the string test [61]. The isolates were inoculated onto blood agar and incubated at 37 °C overnight. After incubation, a single colony was gently stretched with an inoculation loop. The formation of a mucoviscous string measuring ≥5 mm was considered a positive result, indicating hypermucoviscosity.

### 4.4. Whole-Genome Sequencing (WGS) and Bioinformatic Analyses

Genomic DNA was extracted from pure colonies cultured overnight on Columbia blood agar at 37 °C using the High Pure PCR Template Preparation Kit (Roche Diagnostics GmbH, Mannheim, Germany) following the manufacturer’s instructions. The sequencing library was constructed using the Nextera XT DNA Library Preparation Kit (Illumina, Inc., San Diego, CA, USA), and paired-end sequencing was performed on an Illumina MiSeq sequencer (Illumina Inc., San Diego, CA, USA). Raw sequencing reads were processed using the Linux-based WGSBAC pipeline (v2.2.3; https://gitlab.com/FLI_Bioinfo/WGSBAC/, accessed on 23 June 2025), according to Linde et al. [62]. Initial quality control of raw data was performed with FastQC v0.11.7. Genome assembly was carried out using the Shovill assembler, which is based on the SPAdes algorithm [63], and assembly quality was assessed with QUAST [64]. Taxonomic classification and contamination screening were performed using Kraken2. 

MLST was performed in silico using the in-house pipeline WGSBAC (https://gitlab.com/FLI_Bioinfo/WGSBAC, accessed on 2 December 2025) in combination with mlst v2.16.1 (https://github.com/tseemann/mlst, accessed on 2 December 2025), applying the species-specific scheme for *Klebsiella* from PubMLST [65]. 

Virulence-associated genes were detected using ABRicate coupled with the Virulence Factor Database (VFDB) [66]. AMR genes were identified with ABRicate alongside the Comprehensive Antibiotic Resistance Database (CARD) [67], ResFinder [68], and the NCBI’s AMRFinderPlus tool [69]. Plasmid replicons were detected using ABRicate against the PlasmidFinder database [70]. To comprehensively characterise the isolates, all *K. pneumoniae* genomes were analysed using Kleborate v3 [71], a genotyping tool specifically developed to screen genome assemblies of *K. pneumoniae* and its species complex. Core-genome single-nucleotide polymorphisms (cgSNPs) were determined using Snippy v4.6.0 (https://github.com/tseemann/snippy, accessed on 24 May 2026), employing *K. pneumoniae* HS11286 (GCF_000240185.1) as the reference genome. A phylogenetic tree was generated with RAxML v8.2.12 [72] from the cgSNP alignment and subsequently visualised in Microreact [73].

## 5. Conclusions

This study highlights the genetic diversity of *K. pneumoniae* from companion animals, including the detection of human-associated STs and globally recognised high-risk clones associated with nosocomial infections such as ST147. Although overall antibiotic susceptibility was high, the detection of carbapenem resistance in two isolates and colistin resistance in one isolate is noteworthy. Genomic analysis revealed the presence of ESBL determinants, such as *bla*_CTX-M-15_, as well as a hypervirulent *K. pneumoniae* strain and plasmids carrying AMR genes. Overall, these findings indicate that companion animals can harbour *K. pneumoniae* strains carrying AMR determinants and virulence-associated genes. This underscores the need for strengthened regulation of antimicrobial use, comprehensive surveillance programs, and effective infection-control measures in both human and veterinary healthcare to prevent the spread of *K. pneumoniae* across animals, humans, and the environment.

## Figures and Tables

**Table 1:** Antibiotic susceptibility profiles of 59 *K. pneumoniae* isolates.

Antibiotic Agent	Antibiotic Class	Tested Concentration Range, µg/mL	Number of Isolates *		
			R	I	S
Ciprofloxacin	Fluoroquinolones	0.25–2	2	1	56
Levofloxacin	Fluoroquinolones	0.5–2	1	0	58
Amikacin	Aminoglycosides	4–32	0	0	59
Colistin	Polymyxin B	1–8	1	0	58
Chloramphenicol	Amphenicols	8–16	2	0	57
Fosfomycin	Phosphonic acids	32–128	3	1	55
Tigecycline	Glycylcyclines (derivative of the tetracycline class)	0.25–4	1	0	58
Trimethoprim/sulfamethoxazole	Sulfonamides	1/19–4/76	4	0	55
Piperacillin	β-lactams	8–16	9	0	50
Piperacillin/tazobactam	β-lactams	4/4–64/4	2	2	55
Cefotaxime	3rd-generation cephalosporins	1–2	2	0	57
Ceftazidime	3rd-generation cephalosporins	1–128	1	1	57
Ceftazidime/avibactam	3rd-generation cephalosporins	1/4–16/4	0	0	59
Ceftolozane/tazobactam	5th-generation cephalosporins	1/4–8/4	1	0	58
Imipenem	Carbapenems	1–8	2	0	57
Meropenem	Carbapenems	0.125–128	0	0	59

* R: resistant, I: intermediate, S: susceptible.

**Table 2:** Plasmid-borne AMR genes identified in *K. pneumoniae* isolates in the current study.

Isolate ID	Host	Plasmid-Borne AMR Gene(s)	No. of AMR Genes	Associated Antibiotic Classes
23Y0015	Horse	*bla*_CTX-M-15_, *bla*_TEM-1_, *bla*_OXA-1_, *sul*1, *sul*2, *aac*(3)-IId, *aad*A2, *aph*(3″)-Ib, *aac*(6′)-Ib-cr5, *cat*B3, *flo*R, *tet*A, *dfr*A12, *qnr*S1	14	β-lactams, sulfonamides, aminoglycosides, phenicols, tetracyclines, trimethoprim, quinolones
23Y0007	Dog	*aad*A2, *aph*(3″)-Ib, *aph*(6)-Id, *bla*_CTX-M-15_, *qnr*S1, *cat*A2, *sul*1, *sul*2, *mph*A, *dfr*A12	10	Aminoglycosides, β-lactams, quinolones, phenicols, sulfonamides, macrolides, trimethoprim
23Y0002	Dog	*bla*_OXA-1_, *aac*(3)-IIe, *aac*(6′)-Ib-cr5, *qnr*B1, *dfr*A14, *tet*A, *cat*B3	8	β-lactams, quinolones, aminoglycosides, trimethoprim, phenicols, tetracyclines
23Y0130	Horse	*aph*(3″)-Ib, *aph*(6)-Id, *sul*2, *dfr*A14	4	Aminoglycosides, sulfonamides, trimethoprim
23Y0206	Cat	*bla*_TEM-1_, *aad*A1, *lnu*(G)	3	β-lactams, aminoglycosides, lincosamides
23Y0198	Dog	*bla*_TEM-1_, *tet*D	2	β-lactams, tetracyclines
23Y0010	Dog	*bla*_TEM-35_	1	β-lactams
23Y0092	Dog	*tet*D	1	Tetracyclines
23Y0191	Dog	*tet*D	1	Tetracyclines

AMR: antimicrobial resistance; No.: number;

**Table 3:** Summary of data on animal hosts, sampling sites, number of isolates, and geographic origin of 64 putative *K. pneumoniae* isolates.

Host	Sampling Site	Number of Isolates	Geographic Origin
Dog (n = 49)	Feces	32	Lower Saxony (n = 7), Berlin (n = 3), Bavaria (n = 6), Hamburg (n = 1), Hesse (n = 4), Baden-Wuerttemberg (n = 5), and North Rhine-Westphalia (n = 6).
	Urine	4	Lower Saxony, Bavaria, North Rhine-Westphalia, and Mecklenburg-Western Pomerania
	Tracheal swab	3	Hesse, Lower Saxony, and Schleswig-Holstein
	Wound swab	2	Schleswig-Holstein and Lower Saxony
	Nasal swab	1	Lower Saxony
	Skin	1	Baden-Wuerttemberg
	Eye	1	Saxony
	Vocal cords	1	North Rhine-Westphalia
	Abdominal abscess	1	Mecklenburg-Western Pomerania
	Intestine	1	Berlin
	Uterus	1	Schleswig-Holstein
	Not reported	1	North Rhine-Westphalia
Horse (n = 11)	Uterus	3	Saxony, Hesse, and Baden-Wuerttemberg
	Feces	2	North Rhine-Westphalia
	Nasal swab	2	Baden-Wuerttemberg, and North Rhine-Westphalia
	Cervix	2	North Rhine-Westphalia
	Wound swab	1	Lower Saxony
	Penis	1	Thuringia
Cat (n = 2)	Feces	1	North Rhine-Westphalia
	Ear	1	Hesse
Chicken (n = 1)	Feces	1	Saxony
Cattle (n = 1)	Nose	1	Bavaria

## Data Availability

All data generated or analysed during this study are included in this published article and its Supplementary Information files. The raw sequencing data have been submitted to the European Nucleotide Archive (ENA) and are available under the project accession number PRJEB106326.

## Supplementary Materials

The following supporting information can be downloaded at: https://www.mdpi.com/article/10.3390/antibiotics15060556/s1. The supplementary Excel workbook contains six worksheets labeled as follows: Table S1 (Coverage and reads), Table S2 (Assembly quality metrics), Table S3 (AST-, MLST-, and AMR genes), Table S4 (Virulence-associated genes), Table S5 (Plasmid analysis), and Table S6 (SNP differences) for *K. pneumoniae* isolates recovered from animals in Germany.
